# Effect of Dietary Xylanase Inclusion on Growth Performance, Nutrient Digestibility, and Digesta Viscosity of Weaned Pigs Fed Wheat–Soybean Meal-Based Diets

**DOI:** 10.3390/ani14223255

**Published:** 2024-11-13

**Authors:** Gabriela M. Galli, Alejandra Forero Salamanca, Keith Haydon, Crystal L. Levesque, Jorge Y. Perez-Palencia

**Affiliations:** 1 Department of Animal Science, South Dakota State University, Brookings, SD 57007, USA; gabi-gmg@hotmail.com (G.M.G.); alejandra.forerosalamanca@sdstate.edu (A.F.S.); crystal.levesque@sdstate.edu (C.L.L.); 2 CJ Bio America Inc., Downers Grove, IL 60515, USA; keith.haydon@cj.net

**Keywords:** digestibility, enzymes, fiber, intestinal health, nursery pigs, xylanases

## Abstract

(1) Background: This study aimed to evaluate the effects of dietary xylanase addition on growth performance, nutrient digestibility, volatile fatty acids, and digesta viscosity at different digestive sites in weaned pigs fed wheat–soybean meal-based diets with reduced metabolizable energy. (2) Methods: A total of 312 weaned pigs (5.1 ± 0.9 kg, 20 ± 2 days of age) were assigned to one of six dietary treatments. The experimental diets were formulated in a three-phase nursery feeding program: phase 1 (d0–d7), phase 2 (d8–d21), and phase 3 (d22–d42). The experimental diets consisted of a wheat–soybean meal-based diet formulated to meet pig requirements (positive control, PC); the PC diet with a reduction of 100 kcal of metabolizable energy (ME) (negative control, NC); and the NC diet with either 900, 1800, 3600, or 7200 units of xylanase. Feed disappearance and body weight were measured at d7, 14, 21, and 42 in the nursery phase. The pen fecal score was assessed daily from d0 to d14 and three times a week from d15 to d28. On d21–d24 of the experiment (12 pigs per day), one pig per pen was selected for sample collection: ileal, cecal, and mid-colon digesta for viscosity and ileal digesta, feces for nutrient digestibility, and feces and cecal digesta for the measurement of volatile fatty acid. (3) Results: The addition of xylanase to the NC diets did not improve pig growth performance (body weight, feed conversion ratio, and average daily gain; *p* > 0.10) during the entire nursery phase. In Week 2 and Week 3, pigs fed xylanase had a lower (χ2 < 0.05) incidence of fecal scores 3 and 4 (diarrhea) than the PC and NC diets. In addition, the apparent total tract digestibility of neutral detergent fiber and acid detergent fiber increased linearly (*p* < 0.1) in response to xylanase addition. Xylanase addition (900 to 7200 U) decreased digesta viscosity in the colon compared to the PC and NC diets. Furthermore, xylanase addition resulted in a lower (*p* < 0.05) concentration of acetic, propionic, butyric, valeric, and total volatile fatty acid in cecal samples compared to PC. The addition of xylanase resulted in greater acetic and valeric acid concentrations in cecal samples compared to the NC group (*p* < 0.10). (4) Conclusions: Xylanase addition can improve nutrient digestibility, particularly at the total tract level, and reduce viscosity in the hindgut, which could be related to decreasing the occurrence of looseness. However, its impact on growth performance was minimal in wheat–soybean meal-based diets with a reduction of 100 kcal of ME.

## Simple Summary

Pigs do not have endogenous enzymes capable of breaking some fiber components (xylans) found in cereal grains such as wheat. The unique capability of xylanase to break the xylan main chain and release bound glucose from the fiber structure makes it a suitable strategy to feed wheat-based diets to pigs, especially after weaning where, in addition to improving digestibility, xylanase can contribute to promote pig heath. We evaluated the effects of increasing levels of xylanase in wheat–soybean meal-based diets with reduced metabolizable energy on the growth performance, nutrient digestibility, fermentation products, and digesta viscosity in weaned pigs. As a result, xylanase did not affect growth indicators, such as body weight, average daily gain or feed efficiency. However, xylanase supplementation led to a lower incidence of diarrhea, especially in the second- and third-week post-weaning. In addition, nutrient digestibility, particularly fiber components, improved linearly, which was associated with a reduction in digesta viscosity in the colon and changes in cecal volatile fatty acid profiles. In conclusion, xylanase supplementation can improve fiber digestibility and reduce digesta viscosity in the hindgut, which could be related with decreasing the occurrence of looseness. However, its effects on growth were not expressive in wheat-based diets with energy reduction.

## 1. Introduction

The use of exogenous enzymes in pig diets is a nutritional strategy that is widely used to improve nutrient digestion and absorption especially when fibrous ingredients are included in the diet. Wheat is a major cereal grain that is produced throughout the world for both human and livestock consumption [1], but it contains anti-nutritional factors (ANFs) and nonstarch polysaccharides (NSPs) which affect pig growth performance, digestibility, and intestinal health. The high concentration of amino acids in wheat compared with corn makes wheat an attractive alternative to corn, but in general, wheat is lower in energy than corn. In addition, the nutrient value of wheat can vary depending on the class (based on hardness, color, and growing season); however, adverse weather, delayed harvests, or improper storage can result in off-quality wheat [1]. In this context, the use of exogenous enzymes can be implemented as a nutritional strategy to improve wheat nutritional value in swine diets, promote intestinal health in critical phases such as weaning, and maximize pig growth performance.

Carbohydrases have been used to target cell wall NSPs in pig diets because NSPs increase the viscosity of digesta in the small intestine, resulting in a reduced digestibility and absorption of nutrients and overall alteration of intestinal physiological functions [2,3]. Within carbohydrases, xylanase catalyzes the endohydrolysis of 1,4-b-D-xylosidic linkages in xylans, releasing oligosaccharides from dietary fiber [4]. In this context, the arabinoxylo-oligosaccharides can promote the fermentation and the growth of beneficial bacteria (*Lactobacillus*, *Bifidobacterium*, and *Faecalibacterium*) which can result in a higher production of short-chain fatty acids, especially butyrate and propionate [5]. In this regard, dietary xylanase addition can improve nutrient digestibility in pig diets by degrading polysaccharides in the cell wall and reducing digesta viscosity [6]. The inclusion of xylanase can be particularly important after weaning, considering the dramatic change in brush-border digestive enzyme activities following weaning [7]. Xylanase inclusion in post-weaning diets can help pigs in transitioning from milk to grain-based diets by increasing digestive capacity and consequently growth performance. In addition, the dietary addition of xylanase has been associated with beneficial effects on the intestinal health of weaned pigs, including improved gut morphology, reduced inflammatory response, the modulation of mucosa-associated microbiota [8,9,10], increased nutrient digestibility [11], and minimized fecal emissions [12]. Xylanase releases shorter arabinoxlan-oligosaccharides that change the availability of substrates for microbial growth [13], which can reduce the number of potentially pathogenic bacteria [14,15].

Pigs do not have endogenous enzymes capable of breaking β-1,4-glycosidic bonds, which are found in xylan molecules as part of the fiber structure of selected cereal grains such as wheat [16]. In this regard, the unique capability of exogenous xylanase to break the xylan main chain and release bound glucose from the fiber structure of wheat makes it a suitable strategy to feed wheat-based diets to pigs. However, the use of xylanase as part of an enzyme blend resulted in inconsistent results (17), which demands its evaluation alone to evaluate the individual effects. Thus, more information on this matter is important in order to provide nutritionists with validated information on the use of exogenous xylanases, particularly accurate energy matrixes, that can be used in practical diet formulation to produce cost-effective diets using wheat. In addition, dose–response studies could help to better characterize the impact of xylanase on pig growth performance, considering the inconsistent responses reported in the literature [17]. The hypothesis tested in this study was that reducing 100 kcal ME/kg feed during the nursery phase would negatively affect growth performance and intestinal health. It was also hypothesized that increasing levels of xylanase addition could mediate some of the negative effects on growth performance and intestinal health with a reduced 100 kcal ME/kg feed. Therefore, this study aimed to evaluate the effects of dietary xylanase addition on growth performance, nutrient digestibility, volatile fatty acids, and digesta viscosity at different digestive sites in weaned pigs fed with wheat–soybean meal-based diets with reduced metabolizable energy (−100 kcal/kg).

## 2. Material and Methods

### 2.1. Animals, Diets, and Experimental Procedures

The experiment was carried out in a wean-to-finish barn at the South Dakota State University Swine Education and Research Facility, located in Brookings, South Dakota, United States. A total of 312 newly weaned pigs, barrows, and gilts (offspring of PIC Cambrough sows and Duroc boars) with an average initial body weight (BW) of 5.1 ± 0.9 kg and 20 ± 2 days of age (values are presented as mean ± standard deviation) were used in a randomized complete block design. Pigs were weaned into 48 pens (2.44 × 1.83 m) and assigned to one of six dietary treatments at weaning, each treatment with 8 pens, 6–7 pigs per pen (4 pens with 6 pigs and 4 pens with 7 pigs), and sex ratios were maintained within BW blocks (4 pens with 3 barrows and 3 gilts and 4 pens with 2 barrows and 5 gilts). All pens contained one dry self-feeder and one nipple waterer to allow access to feed and water ad libitum. The facility operates on mechanical ventilation with temperatures set at 30, 29, 28, 26.5, 25, and 24 °C for weeks 1–6 of the nursery phase.

The experimental diets were formulated in a 3-phase nursery feeding program (Table 1): Phase 1 (d0–d7), Phase 2 (d8–d21), and Phase 3 (d22–d42). Diets were prepared in a single batch for each phase and stored in airtight bins at room temperature to maintain diet integrity. The dietary treatments were (1) positive control (PC): a wheat/soybean meal-based diet formulated to meet pig requirements [18] except for energy (−50 kcal metabolizable energy ME from the requirement to avoid over formulation due to ingredient variation); (2) negative control (NC): PC diet with a reduction of 100 kcal of ME; (3) NC diet + xylanase addition at 900 units; (4) NC diet + xylanase addition at 1800 units; (5) NC diet + xylanase addition at 3600 units; (6) NC diet + xylanase addition at 7200 units.

The xylanase product used in this study is a GH 11 family fungal endo-1,4-b-xylanase produced from *Orpinomyces* (Activity: 30,000 units, CJ Cheiljedang Bio, Seoul, Republic of Korea). Xylanase activity is quantified as the amount of enzyme required to release 1 μmol xylose equivalent from 1.0% xylan (beechwood or sugarcane bagasse) per min at 50 °C and pH 5.5 (or pH 6.5). Xylanase inclusion levels and the matrix (−100 kcal ME) were based on the manufacture’s recommendations, previous literature, and experimental design (equally spaced between treatments). In addition, reduced nutrient levels in NC were selected based on previous work reporting improvement in digestibility and nutrient value with xylanase inclusion [17,19,20].

The daily animal care observations included pig behavior, recording daily room temperature (high and low temperature), checking waterers and feeders, and treating pigs, if needed. Pigs were treated if they exhibited clinical signs of illness, and the treatment dose, product used, date given, pig and pen identification, and reason administered were recorded throughout the experimental period. Pig mortality/removal data were recorded during the experiment, including day, time, pig and pen identification, pig body weight, and the reason for death or removal.

### 2.2. Data Collection

#### 2.2.1. Growth Performance

Pigs were weighed on d0, 7, 14, 21, 35, and 42 of the experiment. Feed disappearance was measured simultaneously with body weight (BW), average daily gain (ADG), average daily feed intake (ADFI), feed conversion ratio (feed/gain ratio), and gain/feed ratio (G:F) were calculated based on the pigs’ BW and feed intake.

#### 2.2.2. Fecal Score

Fecal scoring was assessed daily from d0 to d14 and three times a week from d15 to d28. A trained single person assessed the fecal consistency scale based on the pen unit [21]. There were four consistency categories: score one = firm and shaped, score two = soft and shaped, score three = loose, and score four = watery, where scores of 1 and 2 represented normal feces, and scores of 3 and 4 represented diarrhea. For each pen, a single observer assigned the relative proportion of visible feces within each category as well as the overall pen score [22].

#### 2.2.3. Digesta and Fecal Collection

On d21–d24 of the experiment, one pig per pen was selected (pig with BW closest to the pen average) for sample collection (12 pigs per day and 2 pigs per treatment per day). Euthanasia was conducted by a trained person using the captive bolt gun technique [23]. Upon euthanasia and evisceration, ileal (70 cm above the ileocecal junction), cecal, and mid-colon digesta samples were collected for viscosity measurements. A subsample of the ileal digesta was obtained for nutrient digestibility analysis. In addition, fecal samples and subsamples of cecal digesta were collected to determine volatile fatty acid concentrations. All samples were stored at −20 °C until analysis.

#### 2.2.4. Nutrient and Energy Digestibility

The apparent total tract digestibility (ATTD) and apparent ileal digestibility (AID) of dry matter (DM), crude protein (CP), gross energy (GE), neutral detergent fiber (NDF), and acid detergent fiber (ADF) were calculated according to the indirect evaluation method during Phase II, using celite as the indigestible marker. The indigestible marker was included in the Phase II diets at 0.3% [24]. From d18 to d20, fresh fecal samples were collected once a day from each experimental pen to evaluate the nutrient digestibility and energy. Fecal samples and the collected ileal digesta samples were homogenized, subsampled, dried, and finely ground over a 0.5 mm screen in a centrifugal mill.

The DM concentration in the diets was determined by drying the samples at 102 °C for 24 h in a drying oven and grinding them to pass through a 0.5 mm screen using a mill grinder (Retsch zm 200, ring sieve size: 0.75 mm). The GE concentrations in the diets and fecal samples were analyzed by bomb calorimetry (Parr 6300 calorimeter, Parr Instruments Co., Moline, IL, USA). The CP (method 990.03), crude fat (Ether Extraction, AOAC Official Method 920.39), crude fiber (CF, AOAC Official Method 978.10, 2006), neutral detergent fiber (NDF, JAOAC 56, 1352–1356, 1973), and acid detergent fiber (ADF, AOAC Official Method 973.18 (A-D), 2006) were determined at a commercial laboratory (University of Missouri, Columbia, MO, USA).

To determine the ATTD of the nutrients, the following equation described by Adeola [25] was used:

Digestibility (%) = 100 − [100 × (M_feed_ × C_feces_/M_feces_ × C_feed_)]

where Digestibility refers to ATTD, M_feed_ and M_feces_ represent concentrations of marker compound in feed and feces, respectively; and C_feed_ and C_feces_ represent concentrations of nutrients in feed and feces, respectively.

#### 2.2.5. Viscosity

Digesta viscosity was determined following the procedure described by Hung et al. [26] with minor modifications. The whole digesta viscosity was measured using a rheometer (ATS Rheosystems, Bordentown, NJ, USA) and a viscometer. All measurements were performed at 39 °C to approximate the body temperature of the pigs. The temperature of the tested samples was monitored by platinum resistance thermometer sensors (accuracy of ±0.1 °C) and controlled by a Peltier system. Approximately 15 mL of sample was poured into a rheometer cup, and then the viscometer spindle was dipped into the cup solution for 2 min to obtain thermal equilibrium conditions between the spindle and the digesta. Then, the rheometer spindle was operated at 30 rpm with continued shearing. For the viscometer, 40 mL of sample was poured into a 50 mL conical tube, the viscometer spindle was dipped into the digesta, and the viscometer was operated. Tests for each sample were performed in duplicates.

#### 2.2.6. Volatile Fatty Acid

Volatile fatty acid (VFA) concentrations in the cecal and fecal samples were determined using a gas chromatographic method. Briefly, samples were thawed, and 2 ± 0.1 g samples were taken, diluted with 4 mL distilled water vortexed for 3 min, and left to rest overnight (4 °C). Then, samples were centrifuged 1500× *g* for 5 min, and the upper layers (1 mL) were acidified with 0.17 mL of metaphosphoric acid (25%, *w*/*v*) and 0.13 mL of internal standard (5 mmol, 4-methyl-valeric acid, 277,827, Sigma, St. Louis, MO, USA), vortexed, and left to rest for 30 min (4 °C). The samples were then centrifuged at 3000 g for 15 min. The supernatant was collected and used for VFA determination using a 6890 N Network GC System gas chromatograph (Agilent Technologies, Santa Clara, CA 95051, USA) equipped with a flame ionization detector according to Izuddin et al. [27]. One microliter of the sample was injected at split 1:30 at a temperature of 230 °C. The separation of the VFA profile was determined using a Quadrex 007-10 Series (Quadrex Corp., New Haven, CT 06525, USA) bonded phase-fused silica capillary column (15 m, 0.250 mm internal diameter, 0.25 μm film thickness). The temperature of the column was set at 60 °C and held for 2 min, increased to 100 °C (10 °C/min), increased to 200 °C (20 °C/min), and held for 2 min. Nitrogen gas was supplied as a carrier gas at a rate of 1 mL/min. The temperature of the detector was set at 230 °C. Commercial standards (Sigma-Aldrich, St. Louis, MO, USA) of acetic (45,997), propionic (94,425), iso-butyric (46,935), butyric (19,215), iso-valeric (78,651), valeric (75,054), and caproic (21,529) acids were used as external standards for peak identification. The molar concentration of VFA was identified based on a single point of the internal standard and a calibration curve with external standards.

### 2.3. Statistical Analysis

The UNIVARIATE procedure of SAS (Version 9.4, SAS Inst. Inc., Cary, NC, USA) was used to confirm the homogeneity of variance and analyze the outliers. Data were analyzed in a randomized complete block design using the PROC MIXED procedure in SAS. In the model, dietary treatment was considered as the main effect, and BW categories were the blocking factor with the pen as the experimental unit. Orthogonal polynomial contrasts were used to determine the linear and quadratic effects of increasing xylanase levels in the NC diets. In addition, contrast statements were used to compare dietary treatments, especially the PC vs. NC, PC vs. 900 to 7200, and NC vs. 900 to 7200. For fecal score analysis, the data were analyzed using the PROC FREQ procedure in SAS. For all statistical tests, differences were considered at *p* ≤ 0.05 and tendency 0.05 > *p* ≤ 0.10.

## 3. Results

The analyzed chemical composition of experimental diets used in this study corresponded to the targets in the diet formulations and were within the tolerance of normal variance (Supplementary File Table S1).

### 3.1. Growth Performance and Fecal Score

The effects of dietary xylanase on the growth performance of pigs are presented in Table 2. Considering the overall nursery phase and under the conditions of this study, increasing levels of xylanase addition to wheat–soybean meal-based diets with a reduction of 100 kcal of ME did not improve pig growth performance. The reduction of 100 kcal of ME resulted in lower G:F and higher F:G, especially during the late nursery phase (d35–d42; *p* < 0.05). The PC group had a greater G:F ratio than the xylanase group (900 to 7200) XU per kg) for d0–d7 and d35–d42 (*p* ≤ 0.10). The PC also exhibited a greater feed efficiency than the energy-reduced dietary treatment groups for d35–d42 (*p* ≤ 0.10). No differences were found among treatments for the other phases (*p* > 0.10).

**Figure 1:**
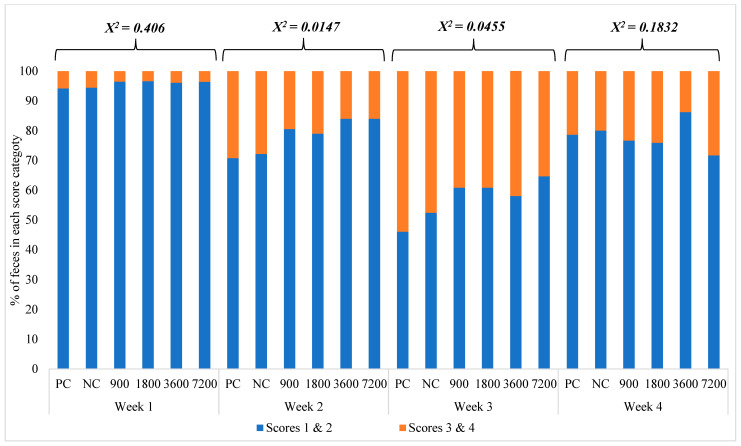
Effects of dietary xylanase addition on fecal scores of weaned pigs fed wheat–soybean meal-based diets ^1^. ^1^ Positive control (PC): a wheat/soybean meal-based diet formulated to meet pig requirements (NRC, 2012) except for energy (−50 kcal ME from the requirement to avoid over formulation due to ingredient variation). Negative control (NC): PC diet with reduction of 100 kcal of ME. Xylanase was included at 30, 60, 120, and 240 g/ton in the negative control diets to create 4 additional dietary treatments consisting of xylanase addition at 900, 1800, 3600, and 7200 U/kg feed, respectively (CJ Bio America INC, 2001 Butterfield Road, Suite 720, Downers Grove, IL 60515, USA).

The effects of dietary xylanase addition on fecal scores of nursery pigs are exhibited in Figure 1. During Week 1, there was a low incidence of diarrhea across all treatment groups. In Weeks 2 and 3, the diarrhea scores generally increased. Pigs fed the PC and NC diets had a greater (χ2 < 0.05) incidence of fecal scores 3 and 4 (diarrhea) than pigs fed xylanase. Fecal scores improved by Week 4 in all pens and treatment groups. Overall, there was a low occurrence (<5% of the pigs) of therapeutic antibiotic treatment during the experimental period without apparent dietary treatment effects. However, at the end of Week 5, the incidence of diarrhea increased across rooms. Fecal samples were submitted to the SDSU Diagnostic Laboratory and tested positive for rotavirus. Even though this is a fairly common virus in nursery pigs within the U.S. swine industry, a secondary *E. coli* infection is common [28]; thus, following a veterinarian’s recommendations, the entire room was treated with neomycin via water for three days. As the treatment occurred in the last week of the trial, previous data or sample collections were not affected.

### 3.2. Nutrient Digestibility and Viscosity

Increasing the levels of xylanase resulted in a linear improvement (*p* < 0.10) in ATTD for NDF and ADF (Table 3). The PC group had greater AID for DM, CP, and GE (*p* < 0.10), as well as greater ATTD for CP, GE, NDF, and ADF, compared to the NC group (*p* < 0.10). In addition, the AID of DM and CP was lower in the xylanase addition diets than in the PC diet (*p* < 0.10). The ATTD of CP and GE was lower in the xylanase addition diets than in the PC diet (*p* < 0.10). The addition of xylanase resulted in a greater AID and ATTD of NDF than NC (*p* < 0.10).

Increasing the levels of xylanase did not affect the digesta viscosity (*p* > 0.10). The addition of xylanase resulted in a lower (−13.40%) viscosity in the colon when compared to PC (*p* < 0.05). Hence, the addition of xylanase resulted in lower viscosity (−10.84%) in the colon than in the NC (*p* < 0.10; Table 3).

### 3.3. Volatile Fatty Acid

The addition of xylanase resulted in a lower propionic acid concentration in feces compared to PC (*p* < 0.10; Table 4). Increasing levels of xylanase addition resulted in a linear decrease (*p* < 0.05) of the proportion (%) of acetic acid in cecal samples, where 1800 U resulted in the highest concentrations (Table 5). The PC group had a greater (*p* < 0.05) proportion (%) of butyric and valeric butyric acids than NC. In addition, xylanase addition resulted in a lower (*p* < 0.05) concentration of acetic, propionic, butyric, valeric, and total VFA in cecal samples compared to PC. The addition of xylanase resulted in greater acetic and valeric acid concentrations in cecal samples compared to the NC group (*p* < 0.10).

## 4. Discussion

This study aimed to evaluate the effects of dietary xylanase addition on growth performance, nutrient digestibility, volatile fatty acids, and digesta viscosity at different digestive sites in weaned pigs fed with wheat–soybean meal-based diets with reduced ME.

In the current study, the growth performance of weaned pigs fed energy-deficient diets (−100 kcal ME/kg) was negatively compromised only from d35 to d42, resulting in no statistical differences across the entire nursery period. These results agree with other work where no difference in growth performance was reported when diet ME was reduced by 100 kcal/kg in corn–wheat–soybean meal diets fed to nursery pigs [20] and corn–soybean meal diets fed to grower pigs [21]. Despite the fact that energy requirements were not met [18], the 3% reduction in ME was not enough to observe quantitative differences in pig growth performance. Torres-Pitarch et al. [17] report in a meta-analysis that the addition of xylanase did not often result in positive effects on growth performance. In contrast, Yang et al. [29] and Petry et al. [30] reported consistent improvements in growth performance when xylanase was included in growing pig diets with different fiber sources/composition. The inconsistency in growth responses to dietary xylanase addition can most likely be related to diet composition, particularly in terms of fiber. For example, experimental diets in Petry et al. [30] included a high-fiber diet with 30% of corn bran without solubles resulting in 21.9% NDF. In the current study, experimental diets were based on wheat and contained between 8.33 and 11.82% NDF (Supplementary File Table S1). In addition, the use of other enzymes in the diets could play a synergistic role that supports xylanase effectiveness. Both Yang et al. [29] and Petry et al. [30] included phytase in their diets. In this study, no other enzymes were used.

Pig growth responses in this experiment did not reflect the observed improvements in nutrient digestibility in PC when compared to NC, demonstrating that improvements in nutrient digestibility are sometimes not translated to enhanced growth performance. A greater digestibility in PC compared with NC could be associated with an increased supply of nutrients in PC pigs that were potentially directed to intestinal health that further improved digestion function. In addition, pigs fed the NC diet performed as good as the PC-fed pigs, while feed intake was the same. This may indicate that the actual energy values of some ingredients used in the diet formulation were higher than the energy values used for formulation. When the NC diet contains enough energy to support the protein deposition of the young pigs, increased digested nutrients in the xylanase-treated group are most likely to be wasted and hence would not be translated to improved growth. In addition, young pigs have underdeveloped digestive systems, which can limit their ability to efficiently absorb nutrients. Their intestinal absorptive capacity is not fully mature, leading to suboptimal nutrient utilization even when digestibility is high. The surface area of the small intestine and nutrient transport mechanisms are still developing in young pigs, which affects overall growth performance (5). Furthermore, gut microbiota plays a significant role in nutrient digestion and absorption. In young pigs, the microbial community is still maturing and may not yet effectively ferment dietary components to maximize nutrient availability (5). As a result, a portion of the absorbed nutrients may be directed toward immune system support and intestinal protein turnover rather than growth performance.

Dietary xylanase addition did improve nutrient digestibility, especially the ATTD of fiber components (NDF and ADF) and the AID of NDF, compared to the NC. The addition of 900 XU appears to have the greatest benefit over nutrient digestion especially considering the ATTD of nutrients. These results could be related to decreased viscosity in the hindgut, as dietary xylanase addition decreased digesta viscosity in the colon. Therefore, the greater digestibility observed in this study may be attributed to the enzyme having better access to the substrates, allowing a greater amount of nutrients to be absorbed. In fact, the addition of exogenous enzymes improved nutrient digestibility by increasing access to endogenous proteolytic, amylolytic, and lipolytic enzymes to nutrients [31]. Baker et al. [19] observed that the addition of 6000 U/kg of xylanase and a reduction of 100 kcal ME/kg feed had a greater DM, GE, CP, crude fiber, and ether extract compared to the negative control (−100 kcal ME/kg) for nursery pigs. Our results also are in agreement with those of a meta-analysis published by Torres-Pitarch et al. [17], who found that DM, GE, and CP digestibility increased with xylanase addition. Soderstrom et al. [32] reported that the inclusion of the multi-enzyme blend (cellulase, xylanase, glucanase, amylase, invertase, and pectinase) increased the coefficients of the apparent total tract digestibility of DM and GE for the wheat and barley-based basal diet. The authors suggested that these results were consistent with the substrates present in the basal diet, specifically arabinoxylans in wheat and β-glucans in barley. Xylanase catalyzes the endohydrolysis of 1,4-b-D-xylosidic linkages in xylans, releasing oligosaccharides from dietary fiber [4], as well as the hydrolysis of the β-1,4-glycosidic bonds of arabinoxylans, which reflects the potential to improve the utilization of fiber by pigs [33].

Weaning stress could be a factor that influenced the pig’s response more than dietary treatments during the first weeks post-weaning, which may explain the overall poor growth performance during the first week and why growth was influenced only in the last period when comparing PC and NC. This could be related to weaning age (20 ± 2 days, below common industry practices in the United States). This occurred due to adjustment in the cycle of the farm. However, in the current study, pig age at weaning was considered as a randomization criterion for pig distribution to pens and consequently to dietary treatments. Thus, all experimental units had similar average weaning age and, therefore, the influence of this factor on pig growth responses was the same across dietary treatment.

Under conditions of reduced dietary energy, growing pigs can regulate their energy balance by increasing daily feed intake [34,35]. In this study, differences in feed intake were not detected between dietary treatments, which may be related to pig age, because weaned pigs (less than 20 kg) have a limited capacity to regulate the feed intake due to gut size compared to older pigs [31,32,33,34,35,36]. Further to the potential impact of age in this study, other studies reporting benefits of xylanase to pig growth used 18.5 kg pigs [29] and 25.43 kg pigs [30]. In both studies, the critical post-weaning period was already over; thus, potential confounding factors of this critical phase were not relevant in those two studies.

When supplemented in nursery pig diets, the benefits of xylanase could be more evident in health-related parameters considering the critical stage of weaned pigs. Moita et al. [10] reported that increasing xylanase (0, 220, 440, 880, 1760 xylanase units per kg feed) improved the intestinal morphology via villus height and reduced the viscosity of jejunal digesta. Similarly to this work, the authors reported that the addition of xylanase did not affect the growth performance of nursery pigs during the overall period. Petry et al. [29] reported health-related benefits following dietary xylanase inclusion in growing pig diets, including improved antioxidant capacity and enhanced gut barrier integrity. In this study, the positive effects of xylanase on pig health, evidenced in the reduction in diarrheal fecal scores, agree with the observation of Boontiam et al. [37] that xylanase addition (45,000, 90,000, and 135,000 U/kg xylanase) decreased the diarrhea rate in weaned pigs. The authors speculated this may have been due to increased feed efficiency through the release of encapsulated nutrients in the plant cell wall and microbiome modulation via the prebiotic effect of the released xylo-oligosaccharides from arabinoxylan hydrolysis. In addition, the NSP skeleton has a water-holding capacity that forms a gelatinous structure surrounding the feed bolus and increases digesta viscosity [38,39]. Xylanase, by lowering the NSP content in the digesta, leaves less fiber accessible in the digestive tract to bind with water and form this gelatinous structure, which explains the lower viscosity in the colon and lower rate of diarrhea reported herein.

The addition of xylanase influenced the volatile fatty acid concentration in the large intestine with potentially positive effects on intestinal health. In particular, NC diets reduced the concentration of butyric acid and total VFA compared to PC, while xylanase supplementation increased their concentration compared to NC. In addition to serving as an energy source for enterocytes, an increased concentration of butyric acid has been associated with improved intestinal morphology, modulation of intestinal permeability, and anti-inflammatory effects [40,41]. This could be correlated with the lower incidence of diarrhea in xylanase-treated pigs. In contrast, Mejicanos et al. [11] observed that the xylanase addition (16,000 U/kg) in a wheat-based diet for weaned pigs reduced butyric acid concentrations. The authors reported that xylanase increased nutrient digestibility in the small intestine, which led to a reduced availability of substrate in the hindgut for fermentation. On the other hand, total VFA production can be influenced by diet composition, especially dietary fiber [42]. In this experiment, soybean hulls were used to reduce energy in NC diets, which could be related with the observed differences between NC and PC for VFA production. However, this was not significantly associated with changes in NDF and ADF digestibility. The evaluation of other fermentation products could have provided valuable information to explain some of these results; however, the sample quantity and lab capabilities were a limitation in this study. Further research in this area is needed.

Finally, evaluating xylanase addition in a wheat-based diet is important because most studies have evaluated the effect of xylanase in corn-based diet. In addition, the responses to xylanase addition in reduced energy diets fed to nursery pigs reported in this work can help determine a specific nutritional matrix of wheat with xylanase addition, which will facilitate diet formulation and improve precision, contributing to sustainable pork production. Further investigation exploring xylanase pathways of xylose metabolism, intestinal permeability, tight junctions, and the microbiota needs to be conducted to better understand the effect of xylanase in pigs and its impact on intestinal health.

## 5. Conclusions

The addition of xylanase improved nutrient digestibility, particularly at the total tract level, and reduced viscosity in the hindgut, which may have contributed to a decrease in the incidence of diarrhea. However, the effects on growth performance were not significant when xylanase was added to wheat–soybean meal-based nursery pig diets with a reduction of 100 kcal of ME.

## Figures and Tables

**Table 1:** Experimental diets formulation and calculated composition (as-fed basis).

Feeds ^4^	Positive Control ^1^			Negative Control ^2,3^		
	Phase I	Phase II	Phase III	Phase I	Phase II	Phase III
Wheat	38.79	48.71	62.23	36.42	47.59	61.08
Soybean meal, 46.5%	20.00	25.00	30.00	20.00	25.00	30.00
Soybean hulls	3.50	2.50	2.50	7.00	5.00	5.00
Dried whey	25.00	15.00	0.00	25.00	15.00	0.00
Fish meal	3.00	0.00	0.00	3.00	0.00	0.00
HP 300	5.00	3.00	0.00	5.00	3.00	0.00
L-Lysine HCl	0.46	0.39	0.29	0.45	0.38	0.28
L-Threonine Pro80	0.19	0.15	0.13	0.19	0.15	0.13
L-Methionine	0.18	0.12	0.07	0.19	0.12	0.07
Soybean oil	1.85	2.44	2.00	0.75	1.10	0.69
Monocalcium phosphate	0.28	0.58	0.53	0.30	0.60	0.53
Limestone	1.10	1.33	1.28	1.05	1.28	1.25
NaCl	0.32	0.45	0.64	0.32	0.45	0.64
Swine vitamin premix ^5^	0.05	0.05	0.05	0.05	0.05	0.05
Swine mineral premix ^6^	0.15	0.15	0.15	0.15	0.15	0.15
Swine larvicide ^7^	0.13	0.13	0.13	0.13	0.13	0.13
Calculated composition						
ME, Kcal/kg	3350	3350	3300	3250	3250	3200
Crude protein, %	23.44	22.83	23.60	23.44	22.92	23.69
Crude fiber, %	3.07	3.03	3.42	4.26	3.90	4.29
Lactose	18.22	10.93	0.00	18.22	10.93	0.00
SID lysine, %	1.50	1.35	1.23	1.50	1.35	1.23
SID Met, %	0.49	0.40	0.36	0.50	0.40	0.36
SID, Met + Cys, %	0.82	0.74	0.71	0.82	0.74	0.71
SID THR, %	0.88	0.79	0.73	0.88	0.79	0.73
SID TRP, %	0.28	0.27	0.27	0.28	0.27	0.27
SID ILEU, %	0.90	0.87	0.86	0.90	0.87	0.86
SID LEU, %	1.51	1.45	1.44	1.51	1.46	1.44
SID VAL, %	0.93	0.90	0.90	0.93	0.90	0.90
SID HIS, %	0.50	0.49	0.52	0.49	0.50	0.52
SID PHE, %	0.92	0.93	0.99	0.92	0.94	0.99
Calcium, %	0.85	0.80	0.70	0.85	0.80	0.70
Phosphorus, %	0.66	0.62	0.57	0.66	0.63	0.57
Dig P., %	0.41	0.36	0.29	0.41	0.36	0.29
Avail. P	0.46	0.41	0.34	0.46	0.41	0.34
Ca/P	1.28	1.28	1.22	1.29	1.28	1.23

^1^ Positive control (PC): a wheat/soybean meal-based diet formulated to meet pig requirements (NRC, 2012) except for energy (−50 kcal ME from the requirement to avoid over formulation due to ingredient variation). ^2^ Negative control (NC): PC diet with reduction of 100 kcal of ME. ^3^ Xylanase was included at 30, 60, 120, and 240 g/ton in the negative control diets to create 4 additional dietary treatments consisting of xylanase addition at 900, 1800, 3600, and 7200 U/kg feed, respectively (CJ Bio America Inc., 2001 Butterfield Road, Suite 720, Downers Grove, IL 60515, USA). ^4^ The feed was formulated according to Phase 1 (d0–d7), Phase 2 (d8–d21), and Phase 3 (d22–d42). ^5^ J & R Distributing Inc. 518 Main Ave, Lake Norden, SD 57248, USA. Minimum provided per kg of diet: Calcium 55 mg, Vitamin A 11,000 IU, Vitamin D3 1650 IU, Vitamin E 55 IU, Vitamin B12 0.044 mg, Menadione 4.4 mg, Biotin 0.165 mg, Folic Acid 1.1 mg, Niacin 55 mg, d-Pantothenic Acid 60.5 mg, Vitamin B16 3.3 mg, Riboflavin mg, 9.9 Thiamine 3.3 mg. ^6^ J & R Distributing Inc. 518 Main Ave, Lake Norden, SD 57248, USA. Minimum provided per kg of diet: Copper 16.5 ppm, Manganese 44.1 ppm, Selenium 0.03 ppm, Zinc 165 ppm. ^7^ Rabon 7.76 oral larvicide premix active ingredient tetrachlorvinphos manufactured for Elanco US, 2500 Innovation Way, Greenfield, IN 46140, USA.

**Table 2:** Effects of dietary xylanase addition on growth performance of weaned pigs fed wheat–soybean meal-based diets ^1^.

Item	PC	NC	Xylanase, XU per kg Feed				SEM	*p*-Value ^2^				
			900	1800	3600	7200		Linear	Quadratic	PC × NC	PC × 900 to 7200	NC × 900 to 7200
Initial BW, kg	5.05	5.01	5.07	5.04	5.06	5.05	0.024	0.502	0.325	0.236	0.837	0.107
Phase, d0–d7												
BW d7, kg	5.34	5.25	5.38	5.18	5.22	5.25	0.063	0.540	0.435	0.312	0.231	0.898
ADG, g	41.41	33.84	43.73	20.32	23.21	28.23	8.404	0.324	0.189	0.528	0.217	0.600
% of pigs that lost weight	11.91	19.35	7.44	16.37	17.26	16.67	6.054	0.759	0.884	0.390	0.599	0.473
ADFI, g	88.55	84.56	94.58	93.11	86.35	86.43	6.386	0.635	0.616	0.662	0.948	0.441
F:G	2.31	2.50	2.62	3.15	3.12	2.71	0.478	0.538	0.035	0.490	0.083	0.238
G:F	0.56	0.44	0.45	0.36	0.27	0.39	0.541	0.238	0.061	0.147	0.009	0.239
Phase, d7–d14												
BW d14, kg	6.44	6.40	6.50	6.29	6.27	6.34	0.085	0.380	0.252	0.725	0.638	0.404
ADG, g	164.0	156.9	159.6	158.7	150.0	156.9	7.921	0.476	0.312	0.532	0.389	0.919
ADFI, g	235.6	245.7	254.7	242.3	238.8	233.6	7.572	0.122	0.237	0.352	0.840	0.300
F:G	1.48	1.56	1.62	1.63	1.59	1.55	0.108	0.930	0.862	0.603	0.448	0.960
G:F	0.69	0.66	0.63	0.65	0.63	0.67	0.039	0.838	0.866	0.543	0.455	0.861
Phase, d0–d14												
ADG, g	99.19	98.94	101.68	89.50	86.61	92.60	5.736	0.276	0.136	0.975	0.402	0.329
ADFI, g	167.1	160.1	174.6	167.7	152.6	160.0	6.008	0.197	0.599	0.412	0.534	0.591
F:G	1.73	1.65	1.75	1.86	1.78	1.78	0.088	0.492	0.272	0.539	0.722	0.165
G:F	0.59	0.61	0.58	0.55	0.57	0.57	0.026	0.219	0.477	0.552	0.627	0.131
Phase, d14–d21												
BW d21, kg	8.56	8.19	8.50	8.22	8.04	8.14	0.224	0.483	0.746	0.243	0.163	0.896
ADG, g	303.6	256.2	285.7	275.7	252.4	255.9	23.72	0.608	0.904	0.166	0.148	0.674
ADFI, g	427.4	391.0	415.0	411.9	371.6	399.6	18.31	0.692	0.692	0.167	0.148	0.679
F:G	1.43	1.67	1.52	1.48	1.57	1.80	0.014	0.279	0.245	0.216	0.237	0.610
G:F	0.71	0.63	0.68	0.69	0.67	0.62	0.042	0.506	0.304	0.204	0.267	0.513
Phase, d0–d21												
ADG, g	167.3	151.3	163.0	151.6	141.9	147.0	10.35	0.441	0.668	0.282	0.158	0.967
ADFI, g	253.9	237.1	254.8	249.1	225.6	239.9	7.891	0.365	0.503	0.139	0.152	0.553
F:G	1.53	1.62	1.61	1.61	1.64	1.75	0.082	0.231	0.681	0.470	0.226	0.724
G:F	0.66	0.63	0.63	0.63	0.62	0.60	0.028	0.383	0.777	0.452	0.253	0.826
Phase, d21–d35												
BW d35, kg	13.32	12.88	13.30	12.89	12.61	12.94	0.434	0.772	0.649	0.479	0.410	0.914
ADG, g	339.8	335.2	343.4	333.7	326.3	342.9	22.13	0.919	0.670	0.885	0.887	0.957
ADFI, g	567.1	553.7	614.3	565.3	525.1	542.4	31.07	0.247	0.676	0.763	0.841	0.817
F:G	1.70	1.65	1.72	1.70	1.62	1.60	0.080	0.322	0.833	0.653	0.630	0.905
G:F	0.61	0.61	0.59	0.61	0.63	0.63	0.029	0.297	0.930	0.976	0.874	0.884
Phase, d35–d42												
BW d42, kg	18.40	18.13	18.35	17.47	17.43	17.68	0.549	0.460	0.388	0.730	0.333	0.518
ADG, g	750.2	725.8	721.4	653.6	688.9	677.7	27.41	0.117	0.123	0.534	0.041	0.366
ADFI, g	1015.4	1095.2	1109.2	1006.2	996.95	1014.5	42.11	0.475	0.684	0.188	0.731	0.156
F:G	1.35	1.51	1.47	1.54	1.46	1.50	0.043	0.139	0.161	0.013	0.006	0.349
G:F	0.76	0.67	0.69	0.65	0.70	0.67	0.022	0.132	0.140	0.007	0.003	0.296
Phase, d0–d42												
ADG, g	317.9	312.5	316.2	295.9	294.5	300.8	12.94	0.140	0.362	0.768	0.333	0.469
ADFI, g	542.5	510.8	558.3	517.4	493.3	509.2	17.13	0.251	0.557	0.198	0.195	0.651
F:G	1.71	1.63	1.73	1.75	1.68	1.71	0.040	0.619	0.456	0.204	0.867	0.075
G:F	0.59	0.62	0.59	0.57	0.60	0.59	0.136	0.628	0.349	0.143	0.712	0.065

^1^ Positive control (PC): a wheat/soybean meal-based diet formulated to meet pig requirements (NRC, 2012) except for energy (−50 kcal ME from the requirement to avoid over formulation due to ingredient variation). Negative control (NC): PC diet with reduction of 100 kcal of ME. Xylanase was included at 30, 60, 120, and 240 g/ton in the negative control diets to create 4 additional dietary treatments consisting of xylanase addition at 900, 1800, 3600, and 7200 U/kg feed, respectively (CJ Bio America INC, 2001 Butterfield Road, Suite 720, Downers Grove, IL 60515, USA). ^2^ Contrast: positive control vs. xylanase addition (900 to 7200 XU/kg); negative control vs. xylanase addition (900 to 7200 XU/kg); positive control × negative control.

**Table 3:** Effects of dietary xylanase addition on nutrient digestibility and digesta viscosity at different digestive sites in weaned pigs fed wheat–soybean meal-based diets ^1^.

Item	PC	NC	Xylanase, XU per kg Feed				SEM	*p*-Value ^2^				
			900	1800	3600	7200		Linear	Quadratic	PC × NC	PC × 900 to 7200	NC × 900 to 7200
Apparent ileal digestibility, %												
DM	80.20	78.65	80.29	78.77	78.63	78.75	0.404	0.138	0.787	0.034	0.019	0.644
CP	86.72	83.27	84.47	84.48	84.43	84.66	1.279	0.586	0.672	0.064	0.087	0.390
GE	84.87	82.95	84.89	82.82	83.86	83.67	0.711	0.857	0.778	0.064	0.123	0.287
NDF	63.09	57.25	67.50	64.30	63.25	66.02	3.439	0.336	0.508	0.288	0.894	0.058
ADF	66.70	65.56	65.75	64.62	69.90	70.29	4.869	0.380	0.907	0.873	0.926	0.704
Apparent total tract digestibility, %												
DM	82.62	82.33	82.60	82.45	82.59	82.48	0.1731	0.754	0.424	0.247	0.503	0.310
CP	85.10	82.90	85.48	83.34	83.60	83.28	0.6418	0.440	0.560	0.020	0.056	0.161
GE	87.15	85.88	87.27	85.42	85.94	85.79	0.4676	0.341	0.779	0.061	0.039	0.667
NDF	65.37	60.00	68.57	65.58	63.94	67.59	1.6144	0.065	0.512	0.024	0.899	0.001
ADF	66.66	61.17	63.61	63.27	64.58	66.52	2.0361	0.072	0.738	0.064	0.213	0.152
Digesta viscosity, Pa. s												
Ileal	134.4	138.3	126.2	122.8	119.0	118.6	9.084	0.159	0.285	0.762	0.350	0.109
Cecum	337.9	331.6	316.7	320.8	321.2	318.4	16.40	0.737	0.788	0.787	0.374	0.506
Colon	1220	1185	1036	1061	1059	1079	57.41	0.536	0.205	0.666	0.036	0.056

^1^ Positive control (PC): a wheat/soybean meal-based diet formulated to meet pig requirements (NRC, 2012) except for energy (−50 kcal ME from the requirement to avoid over formulation due to ingredient variation). Negative control (NC): PC diet with reduction of 100 kcal of ME. Xylanase was included at 30, 60, 120, and 240 g/ton in negative control diets to create 4 additional dietary treatments consisting of xylanase addition at 900, 1800, 3600, and 7200 U/kg feed, respectively (CJ Bio America INC, 2001 Butterfield Road, Suite 720, Downers Grove, IL 60515, USA). ^2^ Contrast: positive control vs. xylanase addition (900 to 7200 XU/kg); negative control vs. xylanase addition (900 to 7200 XU/kg); positive control × negative control.

**Table 4:** Effects of dietary xylanase addition on fecal volatile fatty acid concentrations in weaned pigs fed wheat–soybean meal-based diets ^1^.

Item	PC	NC	Xylanase, XU per kg Feed				SEM	*p*-Value ^2^				
			900	1800	3600	7200		Linear	Quadratic	PC × NC	PC × 900 to 7200	NC × 900 to 7200
VFA, mmol/L												
Acetic C2	35.00	28.53	30.47	35.45	28.65	30.39	2.844	0.94	0.589	0.115	0.175	0.399
Propionic C3	11.20	9.470	9.630	10.22	9.050	9.360	0.882	0.727	0.986	0.172	0.094	0.923
Iso-Butyric iC4	0.660	0.550	0.650	0.480	0.590	0.740	0.131	0.282	0.529	0.583	0.710	0.679
Butyric C4	6.520	4.800	5.030	5.590	4.310	5.040	0.648	0.89	0.797	0.068	0.033	0.795
Isso-Valeric IC5	0.540	0.460	0.590	0.410	0.400	0.600	0.093	0.436	0.213	0.539	0.656	0.672
Valeric C5	1.600	1.270	1.380	1.300	1.420	1.650	0.176	0.140	0.796	0.185	0.301	0.400
Caproic C6	0.110	0.070	0.110	0.130	0.100	0.130	0.026	0.230	0.725	0.333	0.975	0.121
Total VFA	55.62	45.14	47.86	53.58	44.71	47.90	4.244	0.984	0.759	0.088	0.101	0.482
VFA, % of total												
Acetic C2	62.89	63.09	63.36	66.35	62.74	63.79	1.37	0.944	0.698	0.918	0.519	0.530
Propionic C3	20.39	21.15	20.24	19.00	22.29	19.41	1.403	0.703	0.611	0.703	0.986	0.561
Iso-Butyric iC4	1.120	1.230	1.450	0.960	1.220	1.530	0.240	0.403	0.371	0.739	0.543	0.827
Butyric C4	11.58	10.56	10.42	10.18	9.260	10.40	0.713	0.752	0.218	0.317	0.077	0.539
Isso-Valeric IC5	0.920	1.020	1.040	0.820	1.080	1.240	0.179	0.266	0.544	0.702	0.541	0.884
Valeric C5	2.920	2.810	2.930	2.440	2.620	3.360	0.274	0.154	0.123	0.785	0.780	0.931
Caproic C6	0.190	0.140	0.200	0.240	0.320	0.270	0.063	0.185	0.179	0.570	0.528	0.100

^1^ Positive control (PC): a wheat/soybean meal-based diet formulated to meet pig requirements (NRC, 2012) except for energy (−50 kcal ME from the requirement to avoid over formulation due to ingredient variation). Negative control (NC): PC diet with reduction of 100 kcal of ME. Xylanase was included at 30, 60, 120, and 240 g/ton in negative control diets to create 4 additional dietary treatments consisting of xylanase addition at 900, 1800, 3600, and 7200 U/kg feed, respectively (CJ Bio America INC, 2001 Butterfield Road, Suite 720, Downers Grove, IL 60515, USA). ^2^ Contrast: positive control vs. xylanase addition (900 to 7200 XU/kg); negative control vs. xylanase addition (900 to 7200 XU/kg); positive control × negative control.

**Table 5:** Effects of dietary xylanase addition on cecal volatile fatty acid concentrations in weaned pigs fed wheat–soybean meal-based diets ^1^.

Item	PC	NC	Xylanase, XU per kg Feed				SEM	*p*-Value ^2^				
			900	1800	3600	7200		Linear	Quadratic	PC × NC	PC × 900 to 7200	NC × 900 to 7200
VFA, mmol/L												
Acetic C2	28.81	21.15	24.84	27.17	20.01	22.49	2.489	0.630	0.816	0.035	0.043	0.378
Propionic C3	11.63	8.86	10.59	10.21	9.120	9.75	0.855	0.984	0.811	0.027	0.047	0.274
Iso-Butyric iC4	0.170	0.150	0.180	0.120	0.150	0.14	0.042	0.717	0.896	0.770	0.678	0.962
Butyric C4	8.310	4.540	6.430	5.560	5.170	6.08	0.908	0.551	0.948	0.005	0.008	0.217
Isso-Valeric IC5	0.160	0.190	0.250	0.160	0.170	0.18	0.053	0.675	0.693	0.608	0.531	0.968
Valeric C5	1.940	0.880	1.730	1.020	1.500	1.420	0.257	0.383	0.455	0.006	0.032	0.069
Caproic C6	0.080	0.040	0.070	0.040	0.060	0.050	0.020	0.946	0.717	0.121	0.142	0.509
Total VFA	51.09	35.81	44.08	44.29	36.34	40.86	4.080	0.977	0.844	0.011	0.020	0.228
VFA, % of total												
Acetic C2	56.12	59.63	56.37	61.25	55.06	53.50	1.576	0.006	0.922	0.123	0.549	0.087
Propionic C3	22.87	25.62	23.95	23.38	26.36	23.52	1.614	0.698	0.700	0.234	0.343	0.469
Iso-Butyric iC4	0.330	0.440	0.420	0.300	0.570	0.280	0.130	0.585	0.448	0.549	0.600	0.759
Butyric C4	16.35	11.51	14.54	12.34	13.50	14.22	1.513	0.387	0.813	0.029	0.066	0.212
Isso-Valeric IC5	0.310	0.550	0.570	0.390	0.629	0.300	0.149	0.319	0.575	0.259	0.283	0.638
Valeric C5	3.860	2.160	3.980	2.250	3.750	3.190	0.519	0.386	0.281	0.025	0.168	0.058
Caproic C6	0.170	0.090	0.170	0.080	0.150	0.100	0.046	0.957	0.545	0.221	0.311	0.485

^1^ Positive control (PC): a wheat/soybean meal-based diet formulated to meet pig requirements (NRC, 2012) except for energy (−50 kcal ME from the requirement to avoid over formulation due to ingredient variation). Negative control (NC): PC diet with reduction of 100 kcal of ME. Xylanase was included at 30, 60, 120, and 240 g/ton in negative control diets to create 4 additional dietary treatments consisting of xylanase addition on at 900, 1800, 3600, and 7200 U/kg feed, respectively (CJ Bio America INC, 2001 Butterfield Road, Suite 720, Downers Grove, IL 60515, USA). ^2^ Contrast: positive control vs. xylanase addition (900 to 7200 XU/kg); negative control vs. xylanase addition (900 to 7200 XU/kg); positive control × negative control.

## Data Availability

The original contributions presented in the study are included in the article; further inquiries can be directed to the corresponding authors.

## Supplementary Materials

The following supporting information can be downloaded at https://www.mdpi.com/article/10.3390/ani14223255/s1, Table S1: Analyzed chemical composition of experimental diets.
