# Supplementary material for: Effect of Dietary Xylanase Inclusion on Growth Performance, Nutrient Digestibility, and Digesta Viscosity of Weaned Pigs Fed Wheat–Soybean Meal-Based Diets
Source: Animals (Basel). 2024 Nov 13;14(22):3255. doi: 10.3390/ani14223255 (PMC11591498; doi:10.3390/ani14223255)
Supplement: Supplementary file 1 [file animals-14-03255-s001.zip › animals-3275360-supplementary.pdf]

Supplementary File Table S1. Analyzed chemical composition of experimental diets.

	Phase I						Phase II						Phase III					
**Nutrient**	**PC**	**NC**	**900**	**1800**	**3600**	**7200**	**PC**	**NC**	**900**	**1800**	**3600**	**7200**	**PC**	**NC**	**900**	**1800**	**3600**	**7200**
DM, %	91.57	91.72	92.13	92.21	91.71	91.92	90.86	90.61	90.75	90.79	90.51	90.50	89.32	89.32	89.34	89.31	89.52	89.54
Crude protein,%	24.49	25.34	24.16	24.88	24.95	24.54	22.57	22.85	22.60	22.71	23.05	23.19	25.86	26.37	25.54	25.91	25.49	25.56
GE, Kcal/kg	3890	3718	3818	3740	3775	3800	3915	3735	3715	3825	3782	3840	3830	3700	3722	3748	3708	3714
Crude Fat, %	2.46	1.51	1.65	1.68	1.42	1.52	2.71	1.43	1.66	1.64	1.71	1.70	2.41	1.28	1.26	1.09	1.07	1.13
Crude Fiber, %	3.77	4.36	3.95	4.33	4.34	3.91	3.01	3.81	3.57	3.68	3.94	4.16	3.14	4.22	4.46	4.18	3.98	4.04
Ash, %	6.64	7.12	7.35	7.53	6.73	7.35	6.84	6.70	6.90	7.86	6.88	6.83	6.09	5.95	5.48	5.46	5.50	5.91
NDF, %	9.16	11.09	8.68	9.18	9.83	9.96	8.33	9.35	10.01	9.09	9.52	10.02	10.68	11.14	11.09	11.33	11.82	11.39
ADF, %	5.60	6.22	5.73	6.21	6.95	6.08	5.57	6.26	5.67	6.11	6.42	6.10	6.87	6.89	7.21	7.61	6.91	7.44
**Total AA, %**																		
Lysine	1.66	1.64	1.62	1.62	1.60	1.62	1.45	1.38	1.39	1.40	1.39	1.44	1.51	1.47	1.48	1.43	1.46	1.43
Methionine	0.47	0.49	0.49	0.50	0.48	0.49	0.41	0.39	0.42	0.37	0.41	0.40	0.45	0.43	0.39	0.39	0.41	0.45
Threonine	0.98	0.99	0.99	1.04	1.01	0.95	0.90	0.91	0.90	0.89	0.84	0.84	0.92	0.96	0.92	0.88	0.94	0.96
Isoleucine	1.05	1.09	1.06	1.11	1.05	1.07	1.00	0.99	0.97	0.97	0.95	0.95	1.07	1.15	1.09	1.07	1.14	1.12
Leucine	1.73	1.76	1.70	1.79	1.69	1.74	1.63	1.61	1.59	1.58	1.57	1.57	1.77	1.81	1.75	1.76	1.85	1.84
Phenylalanine	1.10	1.09	1.02	1.09	1.05	1.09	1.11	1.10	1.08	1.00	1.07	1.08	1.22	1.21	1.20	1.21	1.27	1.27
Histidine	0.57	0.58	0.54	0.58	0.55	0.56	0.57	0.57	0.56	0.51	0.55	0.55	0.62	0.67	0.62	0.62	0.65	0.65
Arginine	1.32	1.30	1.28	1.29	1.32	1.29	1.35	1.34	1.32	1.34	1.28	1.31	1.47	1.50	1.42	1.45	1.52	1.50
Tryptophan	0.27	0.26	0.27	0.27	0.26	0.25	0.23	0.24	0.24	0.23	0.24	0.24	0.24	0.26	0.25	0.26	0.25	0.26
Valine	1.15	1.17	1.12	1.19	1.13	1.15	1.10	1.09	1.07	0.99	1.05	1.05	1.19	1.28	1.18	1.19	1.25	1.24
Glutamic Acid	5.15	5.01	4.65	4.94	4.88	4.95	5.24	5.09	4.98	4.76	5.03	5.11	5.98	6.15	5.94	5.92	5.99	5.98
Proline	1.61	1.56	1.43	1.51	1.53	1.50	1.60	1.56	1.51	1.43	1.53	1.57	1.81	1.87	1.82	1.79	1.83	1.84
Glycine	1.02	1.03	0.98	1.02	1.01	0.97	0.95	0.96	0.94	0.87	0.93	0.93	1.05	1.12	1.05	1.06	1.11	1.10
Alanine	0.98	1.01	0.98	1.02	0.98	0.96	0.89	0.89	0.88	0.81	0.86	0.86	0.97	1.04	0.97	0.97	1.03	1.02
Cysteine	0.41	0.40	0.39	0.41	0.37	0.38	0.41	0.39	0.40	0.35	0.40	0.38	0.45	0.47	0.44	0.44	0.46	0.46
Tyrosine	0.72	0.67	0.60	0.63	0.58	0.71	0.70	0.69	0.69	0.61	0.68	0.72	0.78	0.87	0.71	0.75	0.75	0.73

[1]Xylanase was included at 30, 60, 120, and 240 g/ton in negative control diets to create 4 additional dietary treatments consisting of xylanase supplementation at 900, 1800, 3600, and 7200 U/kg feed, respectively (CJ Bio America INC, 2001 Butterfield Road, Suite 720, Downers Grove, IL 60515).
